# Endogenous TasiRNAs Mediate Non-Cell Autonomous Effects on Gene Regulation in *Arabidopsis thaliana*

**DOI:** 10.1371/journal.pone.0005980

**Published:** 2009-06-19

**Authors:** Rebecca Schwab, Alexis Maizel, Virginia Ruiz-Ferrer, Damien Garcia, Martin Bayer, Martin Crespi, Olivier Voinnet, Robert A. Martienssen

**Affiliations:** 1 Cold Spring Harbor Laboratory, Cold Spring Harbor, New York, United States of America; 2 Institut des Sciences du Végétal, CNRS UPR2355, Gif-sur-Yvette, France; 3 Institut de Biologie Moléculaire des Plantes, CNRS UPR2353, Université Louis Pasteur, Strasbourg, France; Ecole Normale Superieure, France

## Abstract

**Background:**

Different classes of small RNAs (sRNAs) refine the expression of numerous genes in higher eukaryotes by directing protein partners to complementary nucleic acids, where they mediate gene silencing. Plants encode a unique class of sRNAs, called trans-acting small interfering RNAs (tasiRNAs), which post-transcriptionally regulate protein-coding transcripts, as do microRNAs (miRNAs), and both sRNA classes control development through their targets. TasiRNA biogenesis requires multiple components of the siRNA pathway and also miRNAs. But while 21mer siRNAs originating from transgenes can mediate silencing across several cell layers, miRNA action seems spatially restricted to the producing or closely surrounding cells.

**Principal Findings:**

We have previously described the isolation of a genetrap reporter line for *TAS3a*, the major locus producing AUXIN RESPONS FACTOR (ARF)-regulating tasiRNAs in the *Arabidopsis* shoot. Its activity is limited to the adaxial (upper) side of leaf primordia, thus spatially isolated from ARF-activities, which are located in the abaxial (lower) side. We show here by *in situ* hybridization and reporter fusions that the silencing activities of ARF-regulating tasiRNAs are indeed manifested non-cell autonomously to spatially control ARF activities.

**Conclusions/Significance:**

Endogenous tasiRNAs are thus mediators of a mobile developmental signal and might provide effective gene silencing at a distance beyond the reach of most miRNAs.

## Introduction

**Figure 1:**
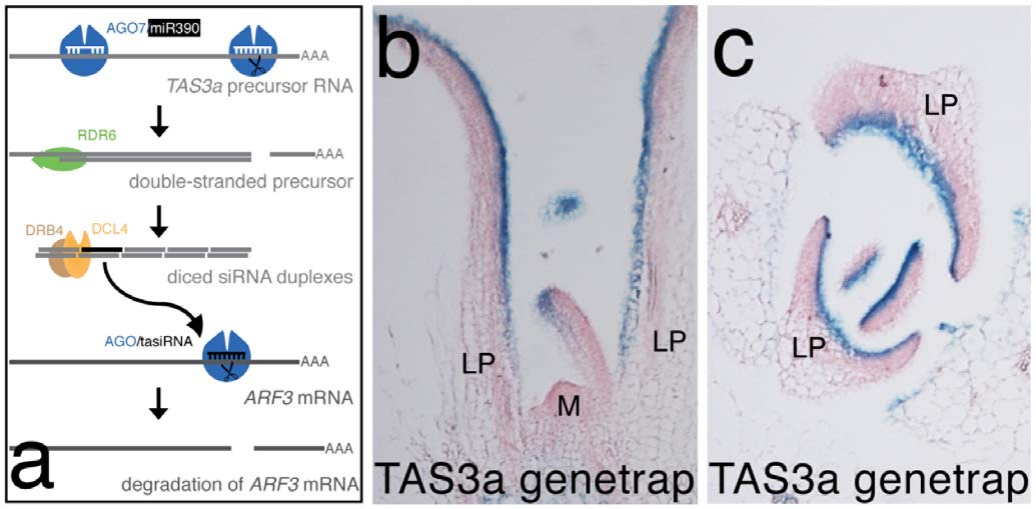
A genetrap inserted at the *TAS3a* locus reports activity in the adaxial L1 and L2 layers. (a) Schematic of tasiRNA biosynthesis from the *TAS3* locus. Longitudinal (b) and transverse (c) sections through the shoot apex visualize the activity of a genetrap inserted upstream of the *TAS3a* locus (GT19682) in the adaxial domain of leaf primordia. “M” marks the shoot meristems, “LP” young leaf primordia.

Different aspects of development in plants and animals are regulated by endogenous sRNAs, most of which belong to the class of miRNAs [1]. Plant genomes in addition encode another group of sRNAs called tasiRNAs, which are also involved in gene silencing, but whose biosynthesis is distinct from that of miRNAs [2]. miRNAs originate from longer single-stranded precursor RNAs, which are directly processed into functional sRNA duplexes by a Dicer endonuclease, DICER-LIKE 1 (DCL1) in *Arabidopsis*. tasiRNA synthesis, however, first requires functional miRNAs, which bind to and trigger cleavage of a non-coding *TAS* precursor RNA. Subsequent synthesis of a complementary strand to the *TAS* precursor forms a double-stranded template for Dicer-mediated tasiRNA production. Genetic screens have identified several players required for tasiRNA biosynthesis, including *RNA-DEPENDENT RNA POLYMERASE 6* (*RDR6*), which mediates double-strand formation [3], [4], and *DCL4*, which processes the double-strand in ∼21mer intervals into tasiRNAs [5], [6], [7]. ARGONAUTE 7/ZIPPY (AGO7) and DOUBLE-STRANDED RNA BINDING PROTEIN 4 (DRB4) function specifically in the synthesis of tasiRNAs from the three *TAS3* loci (*TAS3a-c*) with roles in the initial miRNA-binding step and as a partner of DCL4 respectively (Fig. 1a) [8], [9].

tasiRNAs and miRNAs have a similar mode of action: they base-pair to target mRNAs and post-transcriptionally control target mRNA and/or protein accumulation. The use of tasiRNAs, whose synthesis requires a much larger set of components, and not miRNAs might thus be unanticipated. A possible explanation would be the use of tasiRNAs to mediate silencing non-cell autonomously across cell boundaries, since many miRNAs have been reported to perform their repressive function cell-autonomously [10], [11], [12]; tasiRNAs however require DCL4 and RDR6 for their production, which have previously been implicated in the production of siRNAs from transgenes, whose silencing effects are mobile and spread across several cell layers within the plant [13].

Tretter *et al.* have recently generated an artificial transgene-based system to produce siRNAs from a tasiRNA-like precursor and found non-cell autonomous silencing in various tissues mediated by those siRNAs [12].

We have investigated endogenous tasiRNAs from the *TAS3* loci, which function in the determination of leaf shape in *Arabidopsis thaliana* [3], [4] and also in maize where different factors involved in tasiRNA biosynthesis have been localized in a polar fashion in leaf primordia [14], [15]. We asked whether tasiRNA-mediated gene silencing involves a mobile developmental silencing signal. Our results suggest that tasiRNAs, like transgene-derived siRNAs, manifest their effects non-cell autonomously, and thus propose a specialized role for tasiRNAs in the post-transcriptional control of developmental regulators, which distinguishes itself from that of miRNAs by the range of silencing activities.

## Results and Discussion

### Accumulation of *TAS3* precursor RNA is restricted to the adaxial L1 and L2 layers in leaf primordia

Mutations in genes encoding the tasiRNA biosynthesis components lead to similar phenotypic consequences that are generally weak, including the slight downward curling of rosette leaves and the precocious initiation of trichomes on the abaxial (lower) side of young leaves, also known as accelerated vegetative phase change [16]. These phenotypes are also observed in a *tas3a* insertion mutant, suggesting that *TAS3a* is the major *TAS* locus involved in leaf development [8].

**Figure 2:**
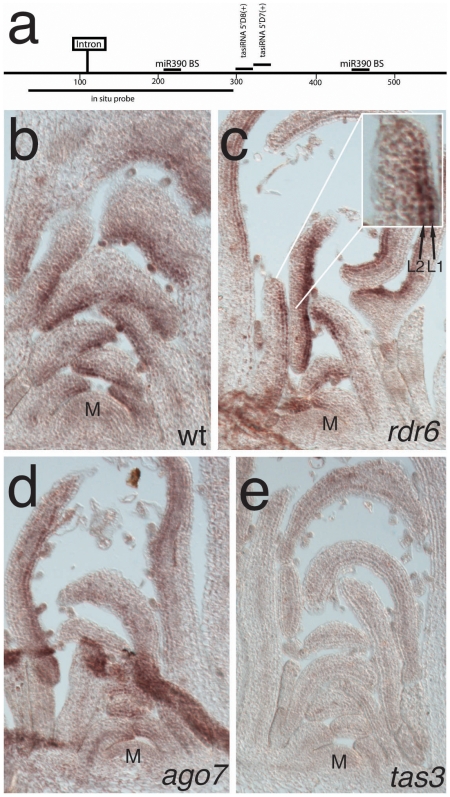
The *TAS3a* precursor accumulates in the adaxial L1 and L2 layer. (a) Schematic of the *TAS3a* precursor transcript. miR390 binding sites (BS) and tasiRNAs complementary to ARF3 and ARF4 transcripts are shown above the bar, the fragment amplified to generate an antisense probe for in situ hybridization is indicated below the bar. *TAS3a* precursor transcripts accumulate exclusively in the adaxial L1 and L2 layer (compare inset), most strongly in the distal tips, as shown by in situ hybridization on wt (b), *rdr6* (c), and *ago7* (d) longitudinal sections through the shoot apex of short-day grown plants, while no signal is detected in *tas3a* insertion mutants (e). “M” indicates the shoot meristem.

We have previously described the isolation of a genetrap reporter line for *TAS3a* whose activity is limited to the adaxial (upper) side of leaf primordia [17] (Fig. 1b, c). In accordance with these genetrap staining patterns, we reproducibly detected *TAS3a* precursor RNA exclusively in the adaxial L1 and L2 layers by *in situ* hybridization on serial longitudinal sections through young wild-type leaf primordia (Fig. 2b). Staining was strongest in the distal part of older primordia (> stage 4) and excluded from the shoot apical meristem. Similar *TAS3a* accumulation patterns were observed in mutants of the tasiRNA pathway *rdr6* and *ago7* (Fig. 2c–d), but no signal was detected in *tas3a* null mutants (Fig. 2e). We were not able to detect *TAS3b* precursor RNA in leaf primordia, neither on sections nor in whole-mount samples (data not shown), consistent with a predominant role for *TAS3a* in generating tasiRNAs after leaf primordia have been initiated. A putative role for *TAS3c* was very unlikely due to its very low capability of producing tasiRNAs [18].

These results indicate that the non-coding RNA precursor transcript of ARF-regulating tasiRNAs accumulates specifically on the adaxial side of leaf primordia, and is refined to the L1 and L2 cell layers within cells which are well separated from the abaxial domain that is specified by the ARF target genes [19].

### ARF3-GUS fusion proteins over-accumulate outside the adaxial L1/L2 layer in the absence of tasiRNA-mediated regulation

Targets of tasiRNAs include the AUXIN-RESPONSE FACTOR proteins ARF2, ARF3/ETTIN and ARF4, which mediate cellular responses to the phytohormone auxin [20], [21], [22]. Both ARF3 and ARF4 promote the specification of the abaxial leaf fate [19]. *ARF4* mRNA is restricted to the abaxial side, while *ARF3* mRNA is detected rather ubiquitously in young leaf primordia by in situ hybridization [19].

**Figure 3:**
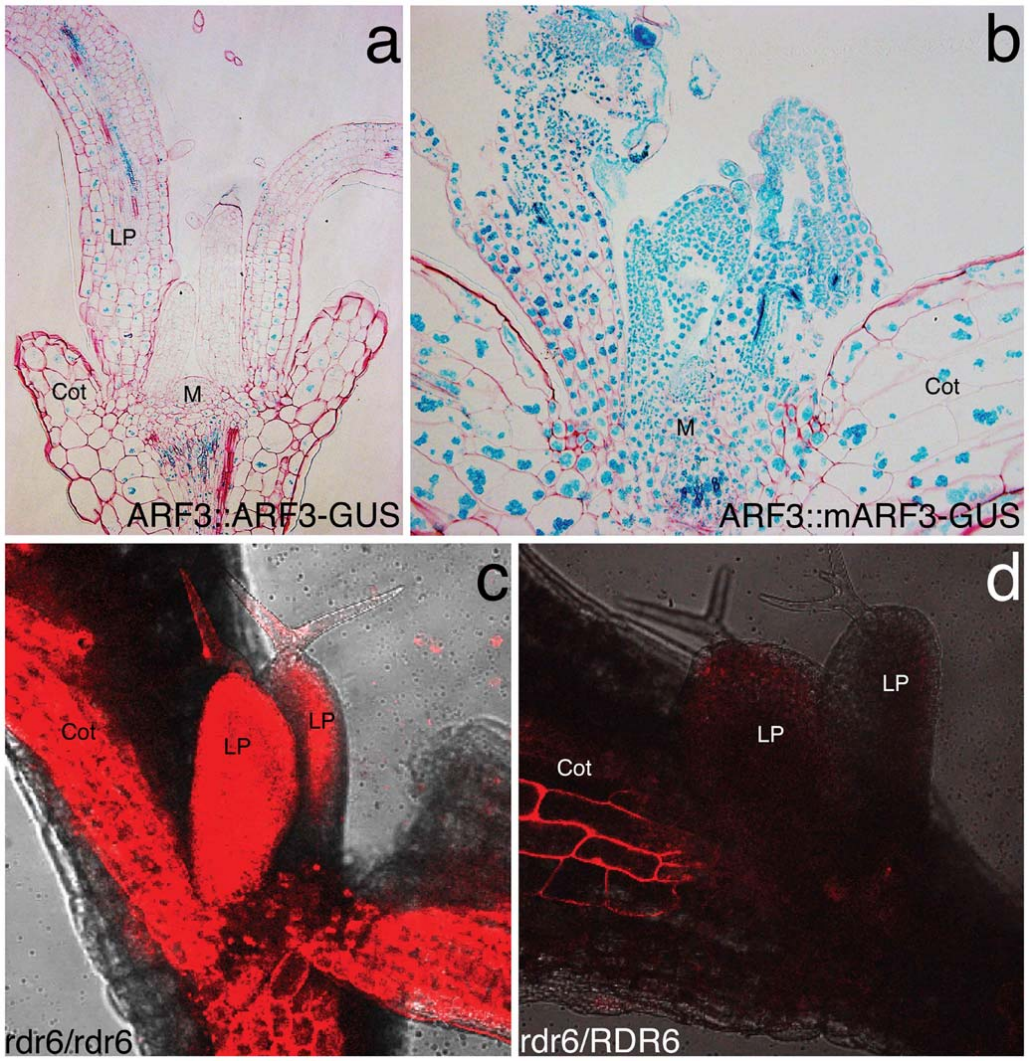
Silencing effects of tasiRNAs are manifested non-cell autonomously. (a) ARF3-GUS protein expressed from its native promoter accumulates only weakly in leaf primordia and is absent from the shoot apical meristem (M). When insensitive to tasiRNA-mediated regulation (mARF3-GUS), much stronger GUS signals are detected throughout primordia as well as the shoot apical meristem with identical staining conditions (b). A ubiquitously expressed tasiRNA-responsive dsRed-sensor is active in most tissues in the absence of tasiRNAs in an *rdr6* mutant background (c), but becomes greatly reduced throughout primordia (LP) and cotyledons (Cot) in the presence of a wild-type *RDR6* copy in heterozygotes, i.e. in the presence of tasiRNAs (d). Exposure times in (c) and (d) were the same.

ARF3-GUS fusion proteins, expressed under their own promoter [23] accumulate only weakly in wild-type leaves (Fig. 3a), however when silent mutations are introduced in the tasiRNA binding sites within the coding region (*mARF3*), ARF3-GUS protein accumulates very strongly not only in the adaxial L1 and L2 cell layer, where the precursor of tasiRNAs is produced, but throughout the leaf and in the meristem (Fig. 3b). These findings imply (1) a potent regulation of ARF3 accumulation mediated by tasiRNAs, and (2) the activity of a non-cell autonomous factor that normally prevents high levels of ARF3-GUS accumulation outside the domain of *TAS3* origin.

### ARF-targeting tasiRNAs exert their silencing effects throughout leaf primordia

To assay for non-cell autonomous spreading of tasiRNA-mediated gene silencing within the shoot apex, we developed a ubiquitously expressed dsRed “sensor” transgene that contains a target site for ARF-regulating tasiRNAs in the 3′UTR with mismatches that resemble the endogenous tasiRNA binding site “A” in the *ARF3* transcript [20]. The absence of dsRed fluorescence in a cell would be indicative of tasiRNA accumulation and activity.

Transgenes were introduced into plants homozygous for the recessive *rdr6-3* mutation [24], in which tasiRNAs are absent such that sensor proteins accumulated near-ubiquitously (Fig. 3c). Three independent strongly expressing lines were backcrossed to the parental L*er* strain to monitor the spatial distribution of tasiRNA-mediated silencing activity in F1 plants, which do express a functional copy of *RDR6* and thus accumulate tasiRNAs. As shown in Fig. 3d, dsRed fluorescence was strongly reduced in young leaf primordia in *rdr6* heterozygotes, both on the adaxial and abaxial sides, suggesting that a silencing factor originating from the *TAS3* transcript was indeed trafficking far out of the adaxial L1 and L2 layers and into most cells of young leaf primordia.

A major concern when silencing transgenes is the RDR6-dependent generation of secondary small RNAs from the primary target (in this case dsRed), which reinforce silencing both locally and in closely surrounding cells. Multiple successive cycles can thus spread the silencing signal throughout the plant [25]. Further, secondary siRNA for many primary miRNA targets are known [26] and can be detected in *ARF3* by deep sequencing [27]. Consistent with an exclusive role of (primary) tasiRNAs, and not secondary siRNAs in dsRed silencing, we never detected complete erasure of dsRed activity in *RDR6* heterozygous plants: fluorescence remained easily detectable in a patch of epidermal cells in the cotyledons (Fig. 3d) and in epidermal cells of root meristems (Supplemental Fig. S1) as well as fully differentiated root cells close to the hypocotyl (not shown).

Therefore we propose that the mobile silencing signal that restricts the accumulation of ARF3 manifests its effects primarily through tasiRNAs. This notion is further supported by our observations that mARF3-GUS over-accumulates ectopically outside the domain of *TAS3*a expression (Fig. 3a and b), since secondary siRNAs originating from the endogenous *ARF3* transcript would be present in both wt- and mutARF3-GUS and silence ARF3-GUS efficiently if they were the critical silencing effector. The endogenous *ARF3* gene may play some role, however, in suppressing the phenotypic effects of ARF3 C- terminal truncations that lack the tasiRNA site [19].

The nature of the mobile component remains unknown. tasiRNAs themselves, due to their small size, serve as an attractive possibility, but the close association of small RNAs with Argonaute proteins needs to be taken into consideration as well, and further research is required to find out if tasiRNAs, or an unstable precursor difficult to detect by in situ hybridization, can directly cross cell boundaries.

Taken together, our data suggests that not only transgene-derived siRNAs [13], but also endogenous tasiRNAs, both of which are produced by DCL4 can manifest their silencing activities non-cell autonomously. The activity of many miRNAs on the other hand, which are mostly produced by DCL1 and often function in a similar developmental context, seems spatially restricted in its silencing potential to the producing or closely surrounding cells [10], [11], [12], [28]. The use of tasiRNAs, which involve a large number of components for their synthesis, rather than miRNAs, might thus directly relate to the range of their biological effects.

## Materials and Methods

### Plant material and growth conditions

All mutants used in this study have been described previously. For *in situ* hybridization, we used mutants in the Col-0 background: *rdr6–15* [29], *zip-1*, referred to as *ago7* throughout the manuscript [21], and *tas3a* [8]. The genetrap insertion line GT19682 is in L*er* background [17], as is the *rdr6–3* allele [24], which was transformed with the tasiRNA sensor construct (see below).

### Transgenic Plants

ARF3::ARF3-GUS and ARF3::rARF3-GUS plants have been described [23]. The tasiRNA sensor was generated by introducing the target site “A” found in ARF3 (same as site “B” found in ARF4) [20] into the 3′UTR of dsRed using the primers 5′-atggcctcctccgagaacg and 5′-taaggatcc**ttcttgaccttgcaagaccct**aatctacaggaacaggtggtggcggccctcg (stop codon of dsRed underlined, target site in bold). The product was placed downstream of the 35S promoter in the binary vector pBIN61 and introduced into L*er* and *rdr6–3* plants with standard techniques.

### GUS staining and histology

GUS staining was carried out as described [30]. Staining were performed for 16 h at 37°C. Samples in Figure 3 were stained in parallel and for the same time. *In situ* hybridizations were carried out as described [30]. Plants were grown in a short day incubator (8 h light, 16 h dark) at 23°C for 30 days, fixed and dehydrated by hand, and embedded into wax with an automated embedding system (Thermo Shandon Excelsior ES Tissue Processor). Sections were 10 µm. The *TAS3a* probe excluded the regions that produce the mature ARF-targeting tasiRNAs (Supplementary Figure S1), and was amplified from *Arabidopsis* Col-0 cDNA with the following oligonucleotides (T3 and T7 sites in capital letters): forward (T3): AATTAACCCTCACTAAAGGgagagaagagctcccatggatgaa, reverse (T7): TAATACGACTCACTATAGGGAGAagagaataatgaaatgcatcatctag. In vitro transcription was carried out with a DIG RNA labeling kit from Roche using T7 RNA polymerase.

### Light microscopy

Histological sections were imaged with a Leica DMRB compound Microscope equipped with Qimaging MicroPublisher 5.0RTV digital camera.

### Confocal imaging

Plants were grown on vertical ½ MS agar plates without antibiotics for 5 days in continuous light at 21°C, mounted in water on glass slides and imaged using a Zeiss LSM510 confocal laser-scanning microscope.

## Supporting Information

Figure S1 Accumulation of the tasiRNA sensor in root meristems. (a) The tasiRNA sensor is detected throughout the root meristem, most strongly in the outer cell layers, in the absence of tasiRNAs in homozygous rdr6-3 mutants, but becomes restricted to the epidermis in the presence of tasiRNAs in RDR6 heterozygotes, i.e. in the presence of tasiRNAs (b).
(0.20 MB TIF)
